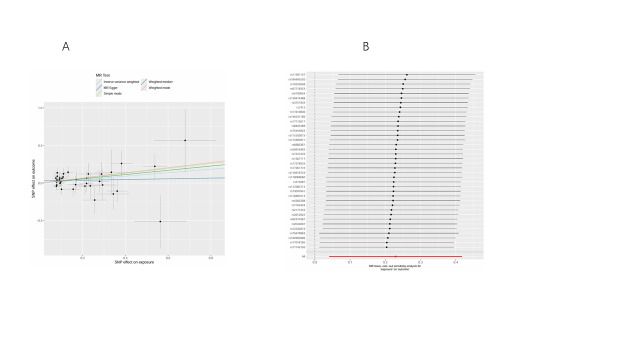# Daily smoking intensity increases lung cancer risk through sphingomyelin and phosphatidylcholine: An in-silico using European population and Mendelian randomization

**DOI:** 10.1097/MD.0000000000048844

**Published:** 2026-05-15

**Authors:** Liancheng Ruan, Han Wu, Yanli Cai

**Affiliations:** a Department of Thoracic Surgery, The Second Affiliated Hospital, Jiangxi Medical College, Nanchang University, Nanchang, Jiangxi, China; b Department of Surgery, Enshi Central Hospital of Tujia and Miao Autonomous Prefecture, Enshi, Wuhan, China.

**Keywords:** cigarettes per day, Mendelian randomization, non-small cell carcinoma, not otherwise specified (NOS), phosphatidylcholine, plasma lipid, small cell lung cancer, sphingomyelin

## Abstract

Lung cancer is a leading cause of morbidity and mortality worldwide, with smoking as a major risk factor. This study investigated the causal relationship between smoking intensity, plasma lipid phenotypes, and lung cancer risk using Mendelian randomization (MR) analysis. Bidirectional MR analysis examined daily cigarette consumption’s impact on non-small cell carcinoma, not otherwise specified (NOS), lung adenocarcinoma (LUAD), lung squamous cell carcinoma (LUSC), and small cell lung cancer (SCLC). Associations between smoking and 179 plasma lipid phenotypes were explored, followed by analysis of lipid groups’ causal links to lung cancer subtypes. Mediation MR estimated the mediation proportion. Smoking intensity showed a significant positive correlation with all lung cancer subtypes (non-small cell carcinoma, not otherwise specified (NOS) [NSCLC-NOS]: odds ratio [OR] = 2.31, 95% confidence interval [CI] = 1.30–4.10; LUAD: OR = 2.99, 95% CI = 1.47–6.10; LUSC: OR = 3.69, 95% CI = 1.39–9.80; SCLC: OR = 3.66, 95% CI = 1.06–12.56). Increased smoking was linked to lower levels of 3 phosphatidylcholine (PC) and higher levels of 2 sphingomyelin (SM) molecules. Bayesian weighted MR revealed 41 plasma lipid phenotypes significantly affected lung cancer risk. PC (16:0_0:0) and SM (d36:2) mediated 1.79% and 3.27% of the risk for NSCLC-NOS, respectively, while SM (d42:2) mediated 2.72% of the risk for SCLC. The study established a causal relationship between daily smoking intensity and the risk of both NSCLC and SCLC, mediated by changes in specific plasma lipid phenotypes. This highlights the critical role of lipid metabolism in smoking-related lung cancer. Understanding the lipid metabolic pathways involved in smoking-induced lung cancer could aid in the development of targeted interventions, potentially reducing the elevated cancer risk associated with increased smoking intensity.

**Highlight**

- Daily smoking intensity causally linked to lung cancer subtypes via MR.
- 41 plasma lipid phenotypes significantly affect lung cancer risk.
- Specific phosphatidylcholine and sphingomyelin levels mediate lung cancer risk.
- Lipid metabolism plays a critical role in smoking-related lung cancer.

## 1. Introduction

Lung cancer remains a principal cause of morbidity and mortality globally, with survival rates in most countries not exceeding 20% even 5 years post-diagnosis.^[1]^ In 2022 alone, there were approximately 2.5 million new cases and over 1.8 million deaths attributed to lung cancer,^[2]^ highlighting the urgent need for in-depth etiological research.

A variety of risk factors contribute to lung cancer, encompassing occupational environments, genetic predispositions, and lifestyle factors, with smoking identified as the most significant.^[3]^ Smoking not only introduces a plethora of carcinogens into the lungs but also promotes inflammatory pathways and DNA mutations, leading to diverse mechanisms of lung cancer pathogenesis.^[4]^ A meta-analysis of Chinese populations underscored the dose-response relationship between smoking exposure and lung cancer risk, demonstrating that cancer mortality escalates with increased smoking (by pack-years),^[5]^ and is even linked to specific oncogenic mutations.^[6]^

Moreover, there is emerging evidence that plasma lipidomics may play a role in modulating the risk of lung cancer influenced by smoking. Lipids, fundamental components of cell membranes and signaling molecules, regulate numerous physiological processes, including cell growth, proliferation, and apoptosis.^[7,8]^ Dysregulation of lipid metabolism has been associated with carcinogenesis, with changes in lipid profiles linked to tumor initiation, progression, and metastasis. Notably, prolonged smoke exposure has been shown to alter pulmonary lipids and lipoproteins in mice,^[9]^ and similar long-term tobacco exposure has been linked to changes in the gene expression profiles related to lipid metabolism in lung adenocarcinoma (LUAD).^[10]^

Interestingly, plasma lipid profiles exhibit heterogeneity among different types of lung cancer, such as elevated levels of specific phosphatidylinositols, phosphatidylcholines (PC), and phosphatidylethanolamines (PE) in non-small cell carcinoma, not otherwise specified (NOS) patients, whereas sphingomyelins (SM) and phosphatidylserines (PSs) are decreased.^[11]^ Conversely, in small cell lung cancer (SCLC), certain PCs and PEs are elevated, while levels of circulating lysoPCs and other PEs are reduced.^[8]^

Despite these advancements, both current and past research reveals an incomplete understanding of how smoking-induced lipid metabolism changes specifically influence NSCLC and SCLC. Additionally, the link between plasma lipids and various lung cancers remains elusive, often obscured by confounders like obesity, diabetes, and cardiovascular diseases in observational studies. To address this, our study applies a Mendelian randomization (MR) framework.^[12]^ MR utilizes genetic variants as instrumental variables (IVs) to discern causal relationships between modifiable exposures, intermediate phenotypes, and disease outcomes. This approach effectively overcomes the confounding and reverse causation issues that are prevalent in traditional observational studies.

In this study, we employ a combination of bidirectional MR and intermediary MR analyses to explore the pathways through which smoking intensity mediates risk by altering plasma lipid phenotypes. Innovatively focusing on the daily intensity of smoking, rather than the more commonly assessed annual consumption, this approach allows for a more detailed exploration of its impacts. These insights are anticipated to elucidate the specific lipid metabolic mechanisms by which smoking influences the pathogenesis of NSCLC and SCLC, thereby offering novel understandings of the lipidomic underpinnings in lung cancer development. The methodologies and results are presented in accordance with the STROBE-MR reporting checklist, accessible at STROBE-MR Checklist (https://jtd.amegroups.com/article/view/10.21037/jtd-23-1645/rc).^[13,14]^

## 2. Materials and methods

### 2.1. Study design

**Figure 1.**
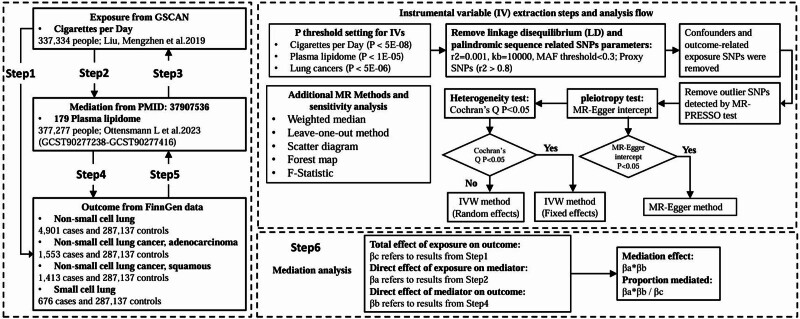
Study design flowchart (Mendelian randomization analysis in European populations). GSCAN = Genome-Wide Association Study and Sequencing Consortium of Alcohol and Nicotine Use, GWAS = genome-wide association study, IVs = instrumental variables, IVW = inverse variance weighted, MR = Mendelian randomization, MR-PRESSO = Mendelian Randomization Pleiotropy RESidual Sum and Outlier, SNPs = single nucleotide polymorphisms.

We conducted a bidirectional MR analysis to examine the causal relationship between daily cigarette consumption and different types of lung cancer. Subsequently, the association between daily cigarette consumption and 179 plasma lipid phenotypes was explored, followed by further analysis of the causal links between plasma lipid groups and lung cancer subtypes. Lastly, the exact mediation proportion was calculated using a mediation MR approach (Fig. 1). Study design flowchart (MR analysis in European populations. All studies were validated through bidirectional MR analysis to confirm the unidirectionality of the results, with a series of sensitivity analyses ensuring result reliability. The study design fulfilled 3 key assumptions: the relevance assumption, ensuring a strong connection between the single nucleotide polymorphisms (SNPs) constructing the IVs and the exposure; the independence assumption, eliminating potential confounding SNPs; and the exclusion restriction assumption, ensuring that the exposure affects the outcome solely through the proposed pathway.

### 2.2. Description of the data source

Table 1 summarizes detailed data sources from genome-wide association studies (GWAS). Phenotypes associated with cigarettes per day were sourced from the GWAS and Sequencing Consortium of Alcohol and Nicotine Use, including 337,334 individuals of European ancestry.^[15]^ Four lung cancer subtypes were obtained from the FinnGen database,^[16]^ comprising non-small cell carcinoma, not otherwise specified (NOS) [NSCLC-NOS] (ncase = 4901), LUAD (ncase = 1553), lung squamous cell carcinoma (LUSC) (ncase = 1413), and SCLC (ncase = 676). Furthermore, data on human plasma lipidomics were sourced from the GWAS catalog database (id: GCST90277238-GCST90277416),^[17]^ covering genome-wide features of 7174 European individuals and encompassing 179 lipid species across 13 lipid categories (Table S1). All datasets were derived from distinct population cohorts to avoid biases from data overlap. Ethical approval and informed consent were obtained for all original studies, and all data are publicly available.

**Table 1:** Details of the studies included in the Mendelian randomization analyses.

Phenotype	Consortium/Author	Ethnicity	Sample size	Year	Data source
Cigarettes per day	GSCAN	European	337,334	2019	https://gwas.mrcieu.ac.uk/datasets/ieu-b-25/
Non-small cell lung cancer (controls excluding all cancers)	FinnGen	European	292,038	2021	https://storage.googleapis.com/finngen-public-data-r9/summary_stats/finngen_R9_C3_LUNG_NONSMALL_EXALLC.gz
Non-small cell lung cancer, adenocarcinoma (controls excluding all cancers)	FinnGen	European	288,690	2021	https://storage.googleapis.com/finngen-public-data-r9/summary_stats/finngen_R9_C3_NSCLC_ADENO_EXALLC.gz
Non-small cell lung cancer, squamous (controls excluding all cancers)	FinnGen	European	288,550	2021	https://storage.googleapis.com/finngen-public-data-r9/summary_stats/finngen_R9_C3_NSCLC_SQUAM_EXALLC.gz
Small cell lung cancer (controls excluding all cancers)	FinnGen	European	287,813	2021	https://storage.googleapis.com/finngen-public-data-r9/summary_stats/finngen_R9_C3_SCLC_EXALLC.gz
179 plasma lipidome	Ottensmann L	European	7174	2023	https://www.ebi.ac.uk/gwas/publications/37907536

GSCAN = GWAS and Sequencing Consortium of Alcohol and Nicotine use.

### 2.3. Selection of genetic instrumental variables

To meet the assumptions of MR studies, the extraction process for all IVs was stringent. The significance threshold for plasma lipid-related phenotypes was set at *P* < 1e−05 to obtain sufficient SNPs. For sample size considerations, the significance thresholds for Cigarettes per Day and the 4 lung cancer phenotypes were set at *P* < 5e−08 and *P* < 5e−06, respectively. All extracted IVs underwent exclusion of linkage disequilibrium (*r*^2^ < 0.001, kb = 10,000) and ensured consistency of allele genes. SNPs with an *F*-statistic < 10 were excluded due to potential bias from weak instrument variables. The LDtrait network tool was utilized to identify and remove confounding SNPs (https://ldlink.nih.gov/?tab=ldtrait).^[18]^

## 3. Statistical analysis

To assess the causal relationship between exposure and outcome, 3 MR methods were applied: inverse variance weighted (IVW), MR-Egger, and weighted median. Given sufficient statistical power in the IVs, IVW is equivalent to two-stage least squares, with smaller errors and more accurate inferences for results interpretation, thus serving as the primary method for inferring causal associations.^[19]^ The MR-Egger method, based on the InSIDE assumption, effectively corrected for the presence of multiple proximal correlations of IVs.^[20]^ When the genetic variation explaining 50% of IVs was achieved, weighted median provided interpretations of causal effects similar to IVW.^[21]^ A causal effect was considered present when the *P*-value of the IVW analysis result was < .05, and the directions of the 3 methods were consistent. To avoid erroneous estimates arising from repeated statistical computations, Bayesian weighted MR is employed to adjust the *P*-values.^[22]^

### 3.1. Mediated MR analysis

The causal effect of daily cigarette consumption on different lung cancer types was defined as the total effect (βc). The causal effect of daily cigarette consumption on 179 plasma lipids was defined as the direct effect (βa). The causal effect between significant plasma lipids and lung cancer outcomes was defined as the direct effect (βb). The mediation effect was calculated using the coefficient product method (βa*βb), and the percentage of mediation effect to the total effect was determined as the mediation proportion.^[23]^

### 3.2. Sensitivity analysis

Sensitivity analysis was conducted to examine the robustness of the results. Cochran *Q* test^[24]^ and MR-Egger intercept test^[21]^ were employed to assess heterogeneity and pleiotropy (*P* < .05), respectively. MR-Pleiotropy RESidual Sum and Outliers (MR-PRESSO) were used to identify horizontal pleiotropy outliers in MR testing.^[25]^ Leave-one-out (LOO) method and scatter plots provided visual outlier detection.

This study was conducted using R 4.3.3, with R packages including TwoSampleMR (version 0.5.11), MRPRESSO (version 1.0), and forestploter (version 1.1.2). Using ChiPlot mapped the heat map (https://www.chiplot.online/).

## 4. Results

### 4.1. Causal relationship between cigarettes per day and lung cancer subtypes

**Figure 2.**
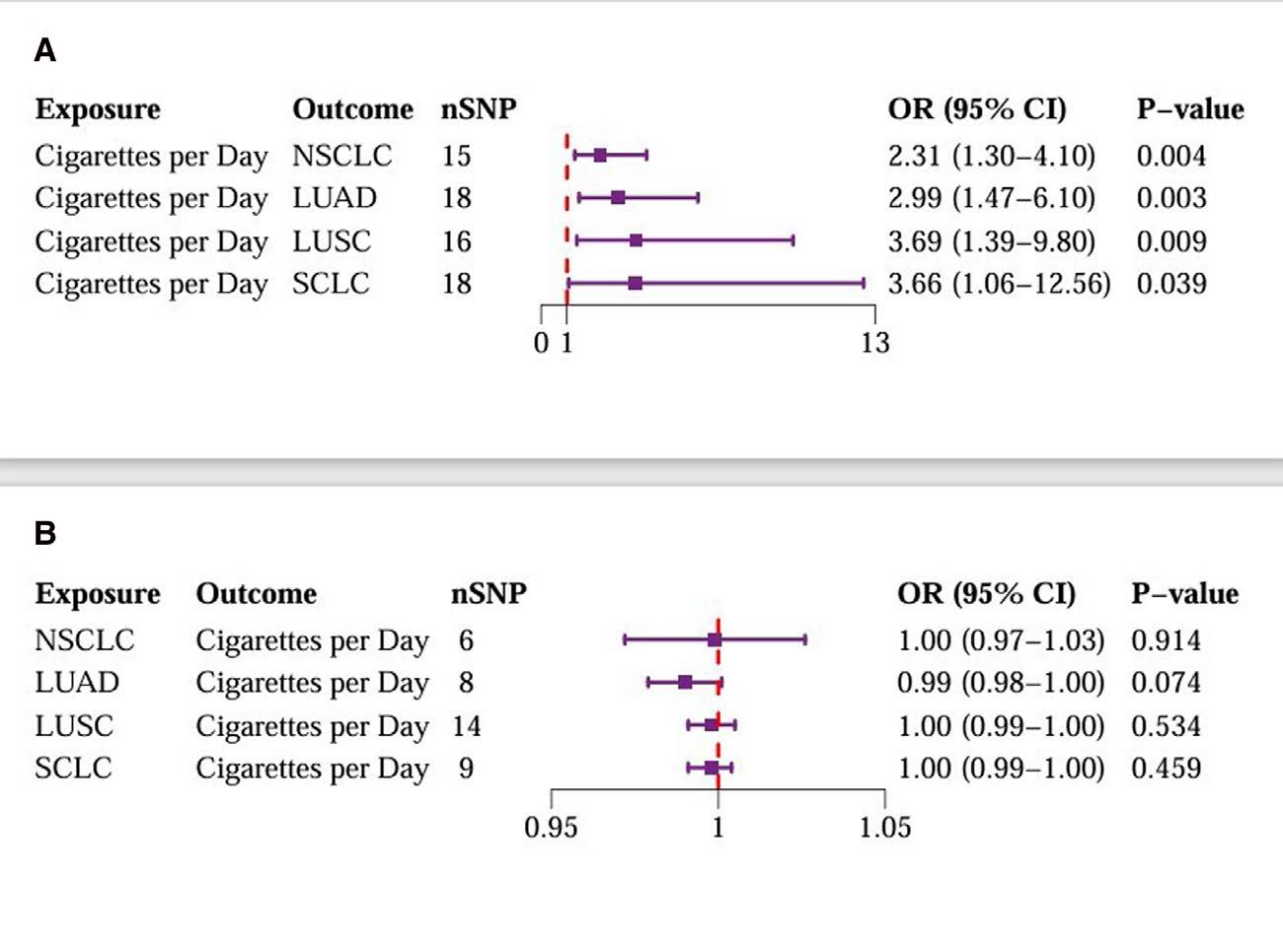
Bidirectional MR analysis of daily smoking intensity and different lung cancer subtypes. (A) Causal effect of daily smoking intensity on lung cancer risk; (B) Causal effect of different lung cancer subtypes on daily smoking intensity. CI = confidence interval, MR = Mendelian randomization, nSNPs = number of single nucleotide polymorphisms, OR = odds ratio.

We conducted bidirectional MR analyses to examine the causal association between daily smoking intensity and 4 distinct subtypes of lung cancer. Our findings revealed a significant positive correlation between daily smoking intensity and all 4 lung cancer subtypes, while lung cancer did not exhibit a significant causal relationship with smoking intensity (Fig. 2A,B). Specifically, NSCLC-NOS (odds ratio [OR] = 2.31, 95% confidence interval [CI] = 1.30–4.10, *P* = .004), LUAD (OR = 2.99, 95% CI = 1.47–6.10, *P* = .003), LUSC (OR = 3.69, 95% CI = 1.39–9.80, *P* = .009), and SCLC (OR = 3.66, 95% CI = 1.06–12.56, *P* = .039) exhibited significant associations with daily smoking intensity. All IVs demonstrated robust instrument strength with *F*-statistics exceeding 10. Heterogeneity and pleiotropy tests did not indicate significant issues, and MR-PRESSO outlier tests successfully identified and excluded outliers (Table S2). The robustness of our results was further confirmed through scatter plots and LOO sensitivity analysis (Figs. S1–S4).

### 4.2. Causal relationship between daily smoking intensity and plasma lipid phenotypes

**Figure 3.**
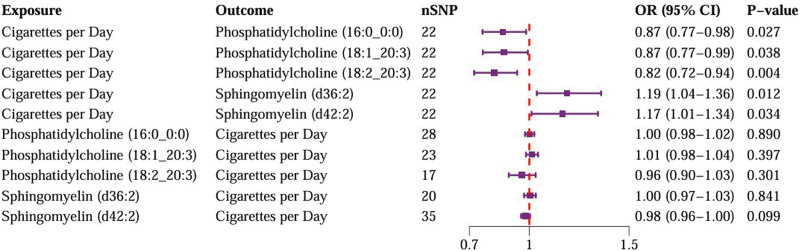
Causal effect between daily smoking intensity and lipids. CI = confidence interval, nSNPs = number of single nucleotide polymorphisms, OR = odds ratio.

Figure 3 demonstrates significant causal relationships between daily smoking intensity and 5 plasma lipid phenotypes, with no significant reverse causal effects observed among these phenotypes. Increased daily smoking intensity was associated with decreased levels of 3 PC molecules: PC (16:0_0:0) levels (OR = 0.87, 95% CI = 0.77–0.98, *P* = .027), PC (18:1_20:3) levels (OR = 0.87, 95% CI = 0.77–0.99, *P* = .038), and PC (18:2_20:3) levels (OR = 0.82, 95% CI = 0.72–0.94, *P* = .004). Conversely, increased daily smoking intensity was associated with elevated levels of 2 SM molecules: SM (d36:2) levels (OR = 1.19, 95% CI = 1.04–1.36, *P* = .012) and SM (d42:2) levels (OR = 1.17, 95% CI = 1.01–1.34, *P* = .034). All IVs exhibited strong instrument strength with *F*-statistics >10. No significant heterogeneity or horizontal pleiotropy was detected, and MR-PRESSO did not identify any outliers (Table S3). The robustness of our results was supported by scatter plots and LOO analysis (Figs. S5–S9).

### 4.3. Causal relationships between plasma lipid phenotypes and lung cancer subtypes

**Figure 4.**
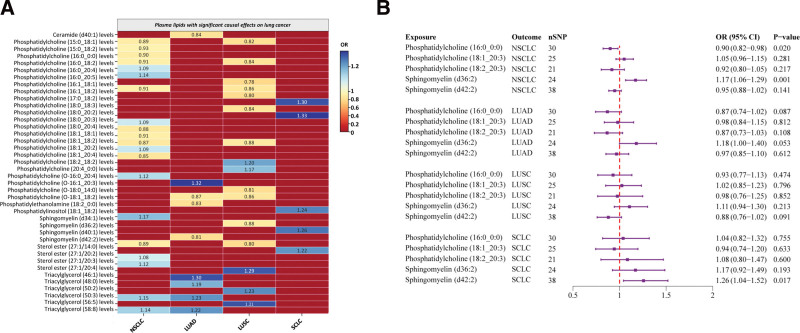
Causal relationship between lipids and lung cancer. (A) 41 plasma lipid phenotypes with significant causal associations with different lung cancer subtypes. (B) Causal effects of 5 smoking-related lipids on lung cancer subtypes. CI = confidence interval, LUAD = lung adenocarcinoma, LUSC = lung squamous cell carcinoma, nSNPs = number of single nucleotide polymorphisms, NSCLC-NOS = non-small cell carcinoma, not otherwise specified (NOS), OR = odds ratio, SCLC = small cell lung cancer.

Following correction of *P*-values using Bayesian weighted MR and eliminating overlapping plasma lipid phenotypes among the various lung cancer subtypes, 41 plasma lipid phenotypes showed significant causal effects on lung cancer risk. Figure 4A provides an overview of the odds ratios that capture the relationships between specific lipid phenotypes and different lung cancer subtypes.

A total of 9 PC subtypes and one sterol ester (SE) demonstrated a negative association with NSCLC-NOS risk. Four PC subtypes, 2 triacylglycerols (TAG), one phosphatidylcholine (O-type, PCO), one SM, and one sterol ester were positively associated with NSCLC-NOS risk. In the case of LUAD, one SE, one P), one ceramide (Cer), and one PE subtypes showed negative correlations, while 3 TAGs and one PCO were positively correlated with LUAD risk. Furthermore, 8 PC subtypes, one SE, one PE, and one SM were negatively associated with LUSC risk. Conversely, one PCO, one PC, and 2 TAG subtypes exhibited positive correlations with LUSC risk. Lastly, 2 PCs, 2 SMs, and one SE showed positive associations with SCLC. No heterogeneity or pleiotropy was detected, and outlier SNPs were excluded, affirming the reliability of the findings (Table S4).

Moreover, further analysis investigated the causal relationship between 5 plasma lipid phenotypes with significant effects on smoking and lung cancer. Figure 4B indicates that PC (16:0_0:0) levels were negatively associated with NSCLC-NOS risk (OR = 0.90, 95% CI = 0.82–0.98, *P* = .020), while SM (d36:2) levels were positively associated with NSCLC-NOS risk (OR = 1.17, 95% CI = 1.06–1.29, *P* = .001). SM (d42:2) levels displayed a significant positive correlation with SCLC risk (OR = 1.26, 95% CI = 1.04–1.52, *P* = .017). None of the 5 plasma lipid phenotypes showed significant associations with LUAD or LUSC. Table S5 confirms strong instrument validity for all IVs (*F*-statistic > 10), with no notable heterogeneity or pleiotropy identified, and MR-PRESSO detected and excluded outliers. LOO sensitivity analyses bolstered the robustness of these findings (Figs. S10–S12). However, discrepancies noted between the MR-Egger and IVW methods for the relationship between PC (16:0_0:0) levels and NSCLC-NOS risk suggest that further validation with larger genetic datasets is required.

### 4.4. Mediated MR analysis

**Figure 5.**
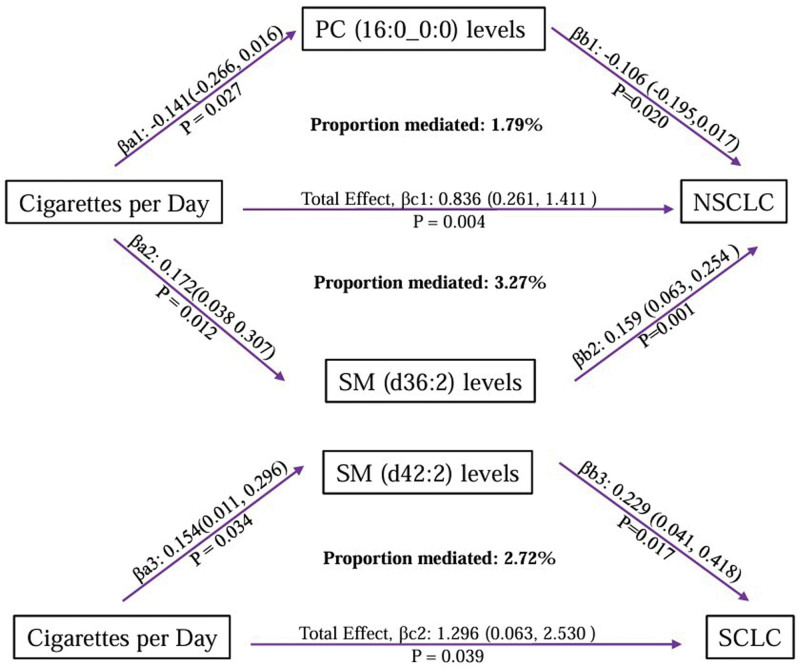
Mediation effect analysis of the relationship between smoking and lung cancer subtypes. NSCLC-NOS = non-small cell carcinoma, not otherwise specified (NOS), PC = phosphatidylcholine, SCLC = small cell lung cancer, SM = sphingomyelins.

Our analysis identified 3 plasma lipid phenotypes that are influenced by daily smoking intensity, subsequently affecting the risk of developing NSCLC and SCLC as depicted in Figure 5 and detailed in Table S6. The modulation of specific lipid levels by daily cigarette consumption appears to mediate the pathogenesis of these lung cancer subtypes. Specifically, a reduction in PC (16:0_0:0) levels and an increase in SM (d36:2) levels, attributable to smoking, collectively mediated 1.79% and 3.27% of the risk for NSCLC, respectively. Moreover, elevated levels of SM (d42:2) were found to mediate 2.72% of the harmful effects of daily smoking on SCLC risk.

## 5. Discussion

This MR study, after analysis of 179 plasma lipid phenotypes, identified 41 that are causally associated with lung cancer risk, among which 5 are significantly linked to daily smoking. Notably, smoking disrupts the metabolism of PC (16:0_0:0) and SM (d36:2), mediated to increase NSCLC-NOS risk by 1.79% and 3.27% respectively. It also raises SCLC susceptibility by influencing SM (d42:2) levels, with a mediation proportion of 2.72%. No lipid phenotypes with mediating effects were found for LUAD and LUSC.

Smoking is the primary risk factor for lung cancer, with 90% of deaths attributable to tobacco exposure.^[26]^ Cigarette smoke generates numerous carcinogens that directly damage DNA and disrupt plasma lipid metabolism.^[27]^ Middlekauff et al^[28]^ observed significant lipid alterations in male tobacco users compared to nonsmokers, with variations in PC and SE, and different changes in free fatty acids in female smokers. In a study by Choi et al,^[29]^ analysis of the metabolomic profile in chronic obstructive pulmonary disease patients identified 8 serum PCs and 3 SMs inversely related to pulmonary quantitative interstitial abnormalities. Our findings corroborate these prior observations by establishing significant causal relationships between 3 PCs and 2 SMs with daily smoking. The underlying mechanisms, potentially related to prolonged tobacco-induced DNA methylation persisting after smoking cessation, remain unclear.^[30]^

Lipid molecules play a pivotal role in regulating cellular membrane integrity, signal transduction pathways, and inflammatory responses. Variations in lipid levels might mirror shifts in the cellular environment, subsequently impacting tumor initiation and progression. In a detailed mass spectrometry analysis, Zhu Z et al^[31]^ compared the plasma lipidomes of 54 lung cancer patients with 14 healthy controls. They noted in LUAD patients an increase in PC and phosphatidic acid (PA) alongside a decrease in PS. In contrast, our study predominantly observed an elevation in TAG and PCO, and reductions in Cer, SE, PE, and phosphatidylinositol. Their findings also highlighted elevated PE and PC, coupled with reduced PA in LUSC, whereas our research identified a significant negative correlation between PC and LUSC. For SCLC, increases in PS and PE and a decrease in PA were noted, aligning broadly with our results. Notably, lipid metabolism exhibited marked variability with discrepancies evident across different ages, genders, and tumor conditions.

Wang G and colleagues highlighted changes in PC (16:0_18:2) levels as one of 9 plasma lipid markers predictive of early-stage lung cancer, a finding consistent with ours.^[32]^ Another analytical study of the lipidomes from 162 NSCLC-NOS patients presented a similar pattern to our MR results, with noticeable declines in the abundance of PC, SM, and PS across both training and validation sets.^[11]^ These observations underscore the inherent heterogeneity of past research, predominantly observational, and highly susceptible to biases from confounding factors such as obesity, diabetes, cardiovascular diseases, and lifestyle dietary habits. Such variability and biases significantly challenge the derivation of definitive causal relationships.

In lung health, PC is essential for maintaining pulmonary homeostasis. Decreased PC levels are linked with idiopathic pulmonary fibrosis and diminished lung function.^[33]^ Patients with idiopathic pulmonary fibrosis show an imbalance between oncogenes and tumor suppressor genes, which may initiate tumorigenesis in fibrotic lung tissues.^[34]^ This suggests how smoking could reduce PC levels and increase NSCLC-NOS risk. SM and its derivatives are involved in signaling that affects ligand-receptor interactions and regulates biological functions.^[35]^ An increase in Cer, a primary breakdown product of SM, can lead to cell necrosis, apoptosis, and disrupt cell proliferation.^[36]^ This elucidates the potential mechanism by which elevated SM levels from regular smoking might elevate the risk for NSCLC and SCLC.

The strength of this MR study lies in its innovative integration of daily smoking, plasma lipidomics, and different lung cancer subtypes to explore potential causal effects. Compared to limited prior metabolomic analyses, our approach expanded sample size and demographics minimizing confounding biases and providing accurate causal inference. Limitations include the study’s focus on the European population, limiting generalizability to non-European groups and a confined number of plasma lipid phenotypes (179 types), precluding causal assessment of other lipids. This study examined daily smoking intensity (cigarettes/day) but not cumulative exposure (e.g., pack-years) due to limited genetic instruments. Future studies are needed to validate these findings more comprehensively and inclusively.

## 6. Conclusion

This study identified a causal relationship between 41 plasma lipid phenotypes and the risk of different subtypes of lung cancer, revealing that 5 of these lipid phenotypes are influenced by daily smoking habits. Through mediatory MR analysis, it was found that daily smoking affects the levels of PC (16:0_0:0), SM (d36:2), and SM (d42:2), thereby increasing the risk for both NSCLC and SCLC. This research highlights the importance of further investigating the impact of environmental factors such as smoking on lipid metabolic pathways, providing new insights for the prevention and diagnosis of lung cancer.

## Acknowledgments

The authors thank the staff and participants of the FinnGen database, GWAS and Sequencing Consortium of Alcohol and Nicotine use (GSCAN), and Open-GWAS database for making the GWAS data publicly available. We thank the coauthors who contributed to this article.

## Author contributions

**Conceptualization:** Liancheng Ruan.

**Data curation:** Liancheng Ruan.

**Project administration:** Liancheng Ruan.

**Supervision:** Yanli Cai.

**Visualization:** Yanli Cai, Han Wu.

**Writing – original draft:** Han Wu.

**Writing – review & editing:** Han Wu.













**Figure:**
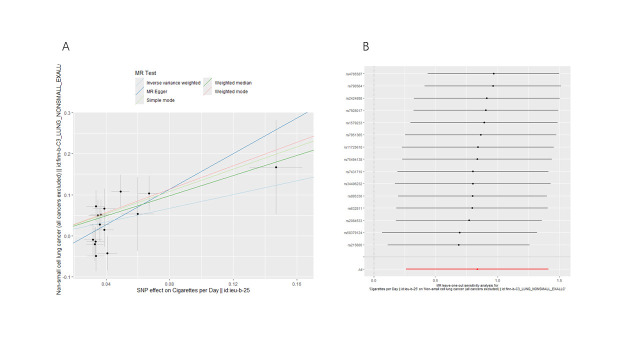


**Figure:**
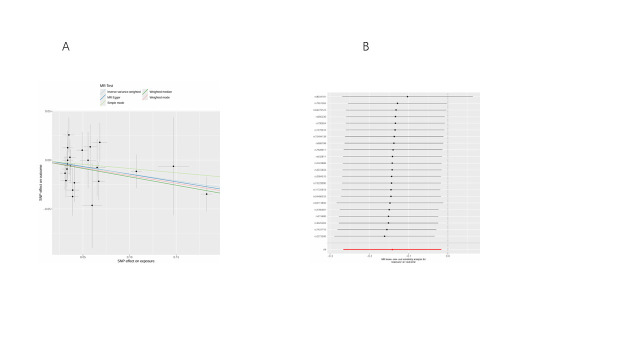


**Figure:**
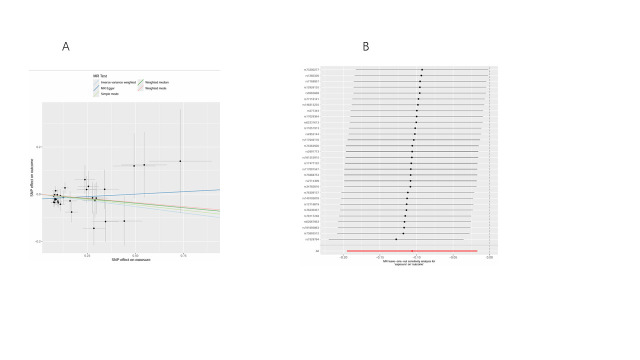


**Figure:**
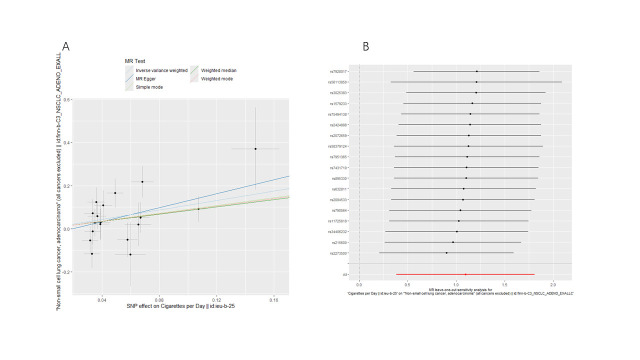


**Figure:**
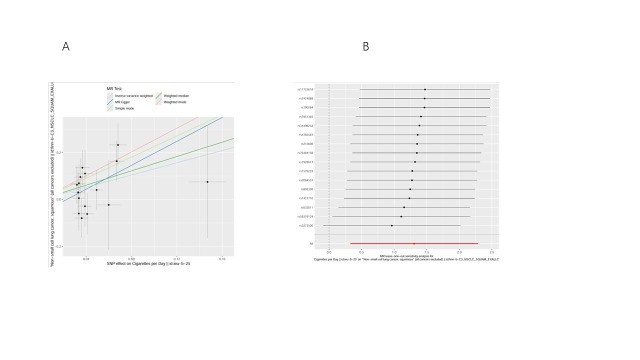


**Figure:**
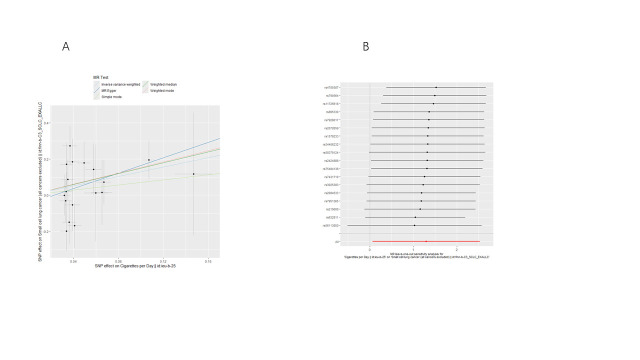


**Figure:**
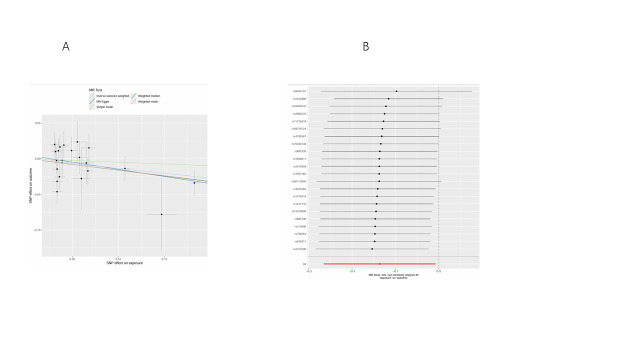


**Figure:**
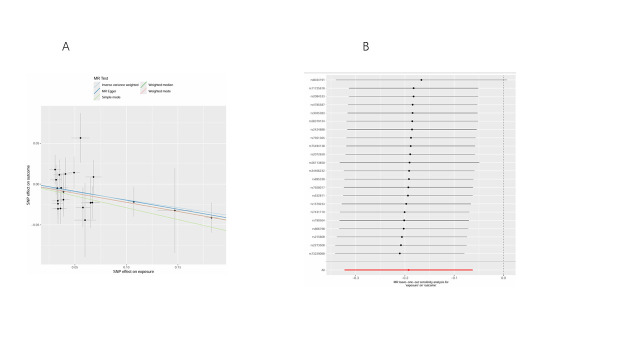


**Figure:**
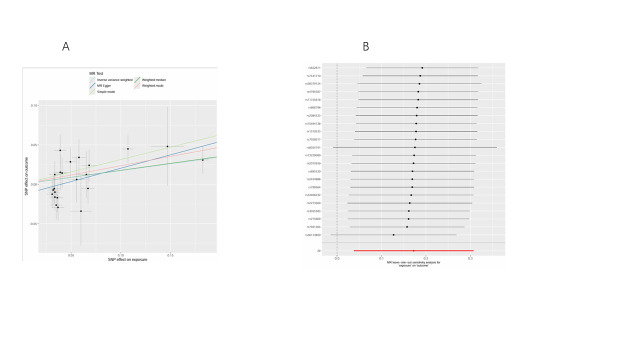


**Figure:**
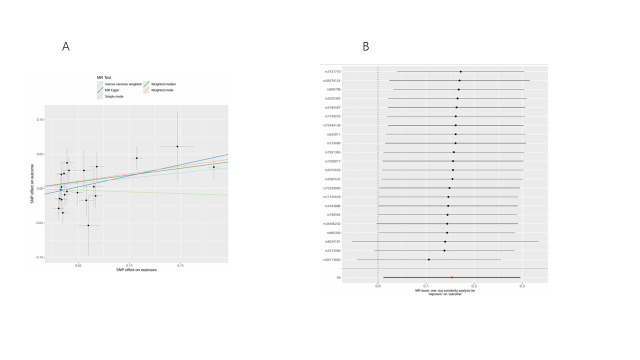


**Figure:**
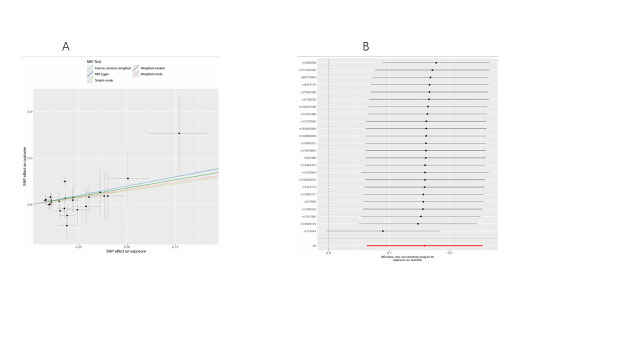


**Figure:**